# The Use of Cefiderocol as Salvage Therapy in an Infant Receiving ECMO and Continuous Renal Replacement Therapy

**DOI:** 10.3390/antibiotics13010037

**Published:** 2023-12-30

**Authors:** Stefania Mercadante, Costanza Tripiciano, Lorenza Romani, Matteo Di Nardo, Gabriella Bottari, Bianca Maria Goffredo, Raffaele Simeoli, Isabella Guzzo, Laura Lancella, Charalampos Antachopoulos, Maia De Luca

**Affiliations:** 1 Infectious Disease Unit, Bambino Gesù Children’s Hospital, IRCCS, 00165 Rome, Italymaia.deluca@opbg.net (M.D.L.); 2 Pediatric Intensive Care Unit, Bambino Gesù Children’s Hospital, IRCCS, 00165 Rome, Italy; 3 Division of Metabolic Diseases and Drug Biology, Bambino Gesù Children’s Hospital, IRCCS, 00100 Rome, Italy; 4 Division of Nephrology and Dialysis, Department of Pediatrics, Bambino Gesù Children’s Hospital, IRCCS, 00165 Rome, Italy; isabella.guzzo@opbg.net; 5 Infectious Diseases Unit, Third Department of Pediatrics, Faculty of Medicine, Aristotle University School of Health Sciences, Hippokration Hospital, 54642 Thessalonik, Greece; bantacho@yahoo.gr

**Keywords:** cefiderocol, pseudomonas aeruginosa, multidrug-resistant, antimicrobial stewardship program, pediatric, intensive care unit

## Abstract

Background: Infections caused by antimicrobial-resistant (AMR) pathogens are increasing worldwide, representing a serious global public health issue with high morbidity and mortality rates The treatment of *Pseudomonas aeruginosa* (PA) infections has become a significant challenge due to its ability to develop resistance to many of the currently available antibiotics, especially in intensive care unit (ICU) settings. Among the very few therapeutic lines available against extensively drug-resistant (XDR)-PA and/or with difficult-to-treat resistance (DTR)-PA, cefiderocol is an injectable siderophore cephalosporin not licensed for use in pediatric patients. There are only a few case reports and two ongoing trials describing the administration of this cephalosporin in infants. Case presentation: This report describes the case of a critically ill 8-month-old girl affected by ventilator-associated pneumonia (VAP) infection complicated by bloodstream infection (BSI) sustained by VIM-producing PA. She was treated with cefiderocol as a salvage therapy during ECMO and CRRT support. Conclusions: In healthcare settings, treating multidrug-resistant, Gram-negative bacteria poses a serious challenge, especially in pediatric patients. Our findings suggest that cefiderocol can be considered as an off-label rescue therapy in selected pediatric cases.

## 1. Introduction

The detection of MDR, XDR, and/or DTRPA isolates has become a common clinical occurrence, especially in ICUs [1].

Although PA strains typically develop resistance due to the interplay of multiple chromosomally encoded mechanisms [2], in recent years, imported resistance mechanisms on mobile genetic elements (such as carbapenemase production) have increased across Europe, as highlighted by the multinational ERACE-PA Surveillance Program. Among 807 carbapenem-resistant PA (CR-PA) isolates collected over the period of 2019–2021 from 17 centers in 12 countries, 33% were phenotypically carbapenemase-positive, and among these, 86% were also genotypically positive, with the most recurrent carbapenemase strain being a metallo-beta-lactamase (MBL) VIM gene-positive strain [3].

In the presence of MBL production, the choice of an appropriate antimicrobial regimen is often challenging due to faster illness onset and faster progression to death in MBL-producing compared to non-MBL-producing CR-PA infections [1]. Moreover, the efficacy of antimicrobial therapy is impaired by the ability of MBLs to hydrolyze and inactivate nearly all β-lactam antibiotics [4].

To date, there are only two therapeutic regimens that show activity against MBL-producing strains: cefiderocol in monotherapy or aztreonam in combination with a beta-lactamase inhibitor belonging to the diazabicyclooctane group, such as avibactam [1].

The optimal cefiderocol susceptibility breakpoint for *P. aeruginosa* is uncertain, with differences in cefiderocol breakpoints: the FDA susceptible breakpoint is ≤1 mg/mL, the Clinical and Laboratory Standards Institute (CLSI) susceptible breakpoint is ≤4 mg/mL, and the European Committee on Antimicrobial Susceptibility Testing (EUCAST) susceptible breakpoint is ≤2 mg/mL. The emergence of PA isolates with MIC above susceptibility breakpoints has been described, making PK/PD optimization crucial in maximizing clinical efficacy and minimizing resistance, also for these novel agents [5]. Currently, particularly among critically ill and/or immunocompromised patients, firm evidence suggests the use of prolonged/continuous infusion for time-dependent antibiotics, such as beta-lactams, to maximize the time that the antimicrobial free fraction remains above the minimum inhibitory concentration (MIC) [6,7,8]. However, extensive PK/PD analyses on the use of cefiderocol in pediatric patients subjected to extracorporeal procedures are still limited [9], and there are no pharmacokinetic data about the use of cefiderocol during pediatric extracorporeal membrane oxygenation (ECMO) and continuous renal replacement therapies (CRRTs).

Here, we describe the case of a critically ill 8-month-old girl affected by ventilator-associated-pneumonia (VAP) infection complicated by bloodstream infection (BSI) sustained by VIM-producing PA, who required ECMO and CRRT support and was successfully treated with cefiderocol.

## 2. Case Report

An 8-month-old girl was transferred from an Albanian hospital to the tertiary care ICU of the Bambino Gesù Children’s Hospital in Rome (Italy) for respiratory failure following viral bronchiolitis, with unremarkable prenatal and perinatal history. Three days after hospital admission, the patient required veno-arterial ECMO support due to severe cardiac dysfunction and pulmonary hypertension. The child also exhibited severe fluid overload associated with anuria, thus requiring CRRT. CRRT was conducted in tandem with veno-arterial ECMO and anticoagulated with heparin.

A CVVHDF modality was used with the following settings: blood flow rate 40 mL/min, reinfusion flow rate 240 mL/h (200 mL/h pre and 40 mL/h post), dialysate flow rate 300 mL/h, and effluent flow rate 25–30 mL/h modulated according to fluid balance and hemodynamic parameters.

Screening for multidrug-resistant organisms performed at ICU admission revealed stool colonization by VIM-producing *P. aeruginosa*. After one week of hospitalization, a VAP with bilateral consolidations, interstitial involvement, and pleural effusion was diagnosed. Bronchoalveolar lavage (BAL) culture showed a high bacterial load of PA (Table 1). Its phenotypic antibiogram highlighted resistance to meropenem (MIC > 16 μg/mL) and ceftazidime/avibactam (MIC > 16/4 μg/mL), intermediate susceptibility to aztreonam (MIC = 1 μg/mL), and susceptibility to amikacin (MIC = 8 μg/mL) and colistin (MIC = 1 μg/mL). A molecular antibiogram confirmed the genotypic detection of VIM metallo-β-lactamase. Combined therapy with ceftazidime/avibactam (50 mg/kg of ceftazidime q8h) plus aztreonam (120 mg/kg/day divided q8h) was promptly started. After one week of treatment, the patient experienced worsening of the clinical condition. BAL cultures still showed significant growth of *P. aeruginosa* with an increasing MIC for aztreonam from 1 to 32 μg/mL and, simultaneously, the blood cultures were positive for the same pathogen (Table 1). Given the evidence of uncontrolled infection, source control was performed by removing central venous and arterial catheters. Susceptibility of PA to cefiderocol was evaluated through the use of an antibiogram. According to the EUCAST defined susceptibility breakpoints for PA [10], this pathogen was considered susceptible to cefiderocol (MIC = 1 μg/mL). Thus, the combined antibiotic therapy previously administered was discontinued and switched to the siderophore cephalosporin. We obtained written parental consent for the off-label use of cefiderocol.

Based on a few studies available on cefiderocol dosing strategies in pediatric subjects, a regimen of 60 mg/kg infused every 8 h (over 3 h) was chosen [9,11,12].

A post-oxygenator blood sample collected on day 3 of treatment resulted in a cefiderocol C*_trough_* value of 51.39 mg/L, achieving the suggested pharmacodynamic target attainment of Cmin/MIC ratio > 4 [13]. The bioanalytical method used to measure cefiderocol blood levels is described in the Section 4.

Over the following days, blood inflammatory markers and the radiological picture quickly improved (Table 1); thus, ECMO assistance and CRRT were discontinued. Lower respiratory and blood cultures tested on the patient resulted negative.

Therefore, cefiderocol was interrupted after 14 days of therapy. During treatment, no elevation in liver tests or other adverse effects were observed. Regarding renal toxicity, the patient was initially supported by CRRT for severe acute kidney injury, as mentioned above. After four days of treatment with cefiderocol, CRRT was discontinued, and renal clearance remained within the normal age-specific ranges throughout the remaining course of therapy.

The patient was discharged from the pediatric ICU after 27 days of hospitalization.

No hospital readmission within 28 days was observed. Three months after discharge, the patient was in good clinical condition, and she was still colonized by VIM-producing PA in her stool.

**Figure 1:**
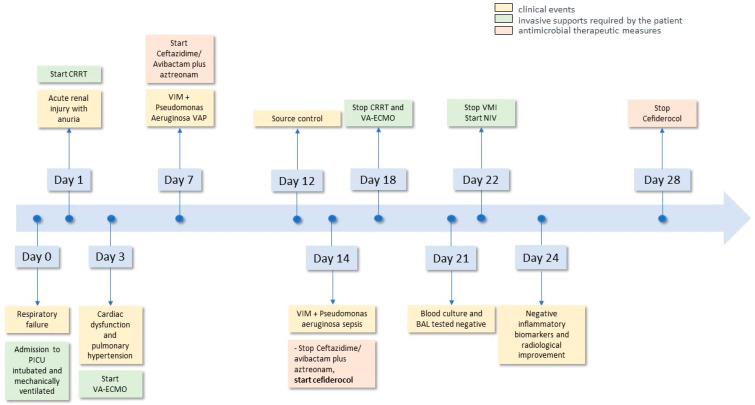
Timeline of the patient’s clinical course.

The patient’s clinical course is summarized in Figure 1.

## 3. Discussion

Data from the European Antimicrobial Resistance Surveillance Network (EARS-Net) for the year 2021 revealed significant variations in the percentages of carbapenem-resistant *P. aeruginosa* within the WHO European Region. The national percentage of carbapenem-resistant *P. aeruginosa* isolates in Italy was reported to be 16.4% [14].

The mechanisms of PA resistance are complex and include mutations in OprD porins, the hyperproduction of AmpCs, the upregulation of efflux pumps, and the production of carbapenemases [2,15]. Carbapenemase-producing PA strains are rising, particularly in Europe, posing a serious problem due to the potential loss of effectiveness of anti-pseudomonal agents, including ceftazidime, cefepime, and piperacillin–tazobactam, as well as the new beta-lactam/beta-lactamase inhibitor combinations such as ceftazidime–avibactam, ceftolozane–tazobactam, and imipenem–relebactam [16]. Aztreonam is capable of withstanding hydrolysis by MBL carbapenemases; however, it is generally susceptible to hydrolysis by serine β-lactamases, including ESBLs, AmpCs, KPC, and OXA-48-like carbapenemases. Since plasmids containing the MBL genome can also harbor genes that encode for several β-lactamases [17], a combination of aztreonam and avibactam provides broad coverage against a wide range of β-lactamase-producing Enterobacteriaceae. However, although a combination of aztreonam and avibactam has been shown to be effective against MBL-producing Enterobacterales [18], its activity against PA is less predictable due to multiple intrinsic and acquired resistance mechanisms that characterize multidrug-resistant PA. Clinical case series describing MBL-PA infections treated with ceftazidime–avibactam combined with aztreonam have reported satisfying results [19,20]. Furthermore, a previous study, aimed at evaluating the time-killing profile of aztreonam and ceftazidime–avibactam in combination against MBL-producing *P. aeruginosa*, showed synergic and bactericidal activity in 80% of tested isolates [21]. Supported by this literature data and since both ceftazidime/avibactam and aztreonam (but not cefiderocol) have obtained European Medicines Agency (EMA) approval for use in pediatric patients [22], the patient described in the presented case report was initially treated with the avibactam/aztreonam combination. Unfortunately, this combined therapy was not effective, probably due to multiple concomitant factors. First of all, sepsis in critically ill patients is responsible for multiple physiological and pathological changes that alter the PK behavior (distribution and clearance) of many drugs including beta-lactams. Moreover, extracorporeal procedures (CRRT and ECMO) may also affect drug clearance [23]. Furthermore, the ceftazidime/avibactam combination was administered by intermittent infusion instead of prolonged/continuous infusion as suggested by the latest real-world evidence that shows better achievement of the desired pharmacokinetic/pharmacodynamic (PK/PD) target by prolonged/continuous infusion [5,24]. Unfortunately, in our case, therapeutic drug monitoring (TDM) for ceftazidime/avibactam and aztreonam was not performed; therefore, we are not aware of whether the drugs blood levels reached the PK/PD target of efficacy. A further possible hypothesis for the therapeutic failure of the avibactam/aztreonam combination could have been the acquisition by PA of further resistance mechanisms (apart from carbapenemase production). The increase in the MIC value for aztreonam from 1 to 32 mg/L during treatment could suggest the occurrence of different resistance mechanisms, such as the overproduction of pseudomonal inducible Amp-Cs [25].

Cefiderocol is an injectable siderophore cephalosporin that binds to iron and uses a “Trojan horse” entry mechanism to penetrate into bacterial cells, overcoming the loss of porin channels and/or the overexpression of efflux pumps. This mechanism of action confers to cefiderocol’s high stability to Ambler classes of β-lactamases and carbapenemases including VIM [26]. Although it has not yet received approval from the EMA for pediatric use, two clinical trials [9,12] are currently ongoing in pediatric cohorts, proposing a dose of 60 mg/kg every 8 h for weight <34 kg and 2 g every 8 h for weight ≥34 kg through a 3 h extended infusion regimen. Considering that the patient described in this case report received both ECMO and CRRT support, dosage modifications were discussed by the clinician’s team. Sequestration of antibiotics in the circuit, increased volume of distribution (Vd), and decreased clearance (CL) are the major PK changes associated with ECMO support, particularly in children, since hemodilution caused by the priming solution is almost twice of patient’s blood volume [23]. Previous studies have shown that cefiderocol is not significantly adsorbed on the oxygenator membrane in the ECMO circuit; therefore, cefiderocol clearance is not expected to be altered in patients with ECMO support, despite the relatively high percentage of drug-free fractions [27]. Concerning the potential impact of the CRRT on the siderophore cephalosporine, previous studies have shown that cefiderocol could be highly removed by a dialysis hemofilter due to its high unbound fraction (50%) and low molecular weight (1000 Da) [28].

Furthermore, other recent studies using in vitro models of CRRT have pointed out that the effluent rate was the significant parameter determining the clearance of cefiderocol, suggesting that the standard dosage for cefiderocol should be used in adults for an effluent flow rate ≥ 4.1 L/h [29], as currently reported in the FDA-approved prescribing information [30]. Considering the high effluent flow rate used in our patient, the dose of cefiderocol proposed in the ongoing pediatric trials was not modified. Therefore, in this case report, the desirable PK/PD target of efficacy for cefiderocol (C_min_/MIC ratio ≥ 4) was achieved. Unfortunately, we were not able to perform multiple sampling (post-hemofilter and effluent waste bag) in order to calculate the contribution of CRRT (%) removal to the total clearance in our patient. However, future evidence will be required to better clarify the impact of extra-corporeal therapies on the removal of siderophore cephalosporines.

In terms of safety, no adverse events were reported in our patient, suggesting that the proposed dosing strategy based on extended infusion may be adequately safe and able to reach the PK/PD target of efficacy.

## 4. Materials and Methods

Cefiderocol plasma levels were determined by using a modified version of our previously published method [31]. Briefly, 100 μL of plasma was spiked with 50 μL of butylparaben (used as an internal standard, IS) and vortexed for 30 s; thereafter, the mixture was extracted with 250 μL of acetonitrile, mixed for 30 s, and centrifuged at 13,000 rpm for 9 min. The supernatant was collected and evaporated under liquid nitrogen flow. Reconstitution was achieved with 100 μL of 2-(N-morpholino) ethanesulfonic acid (MES) buffer. The chromatographic run was realized on a Kinetex^®^ 5 μm EVO C18 150 × 4.6 mm column (Phenomenex, Torrance, CA, USA) thermostated at 25 °C with 1.0 mL/min flow. The analytes were discriminated by means of gradient elution. Mobile phase A consisted of Na_2_HPO_4_∙2H_2_O 0.35% in H_2_O (adjusted to pH 7 with H_3_PO_4_), and mobile phase B was acetonitrile. The total running time was 18 min, and the injection volume was 40 μL. The cefiderocol concentrations were calculated from a linear calibration curve ranging from 5.0 to 200.0 µg/mL. Chromatographic analyses were carried out on an HPLC Agilent 1260 Infinity II system equipped with a quaternary pump, a degassing line, a fluorimetric detector, a column oven, and a cell DAD (diode array detector) (Agilent Technologies, Deutschland GmbH, Waldbronn, Germany). UV detection for both cefiderocol and IS was realized at 260 nm with retention times of 6.73 and 9.49 min, respectively. In order to assess the selectivity of the cefiderocol chromatographic method, six different blank (drug-free) plasma samples were spiked with or without the internal standard (IS) and analyzed to evaluate possible endogenous interferences with the detection of cefiderocol. As reported in Supplementary Figure S1A, blank samples spiked with IS did not show interfering peaks within the cefiderocol retention time. The median signal of these blank samples was below 20% of the LLOQ, thereby ensuring the selectivity of the method. Conversely, Supplementary Figure S1B,C depict chromatograms for calibrator 3 (50 µg/mL) and the patients’ samples, respectively. At a retention time of 6.76 min, we did not observe the presence of endogenous or exogenous interfering peaks. Moreover, to assess the presence of carry-over, IS-spiked blank samples were run in triplicate, following the highest calibration point. According to EMA guidelines, the median signal of these blank samples was less than 20% of the LLOQ and 5% of the IS, confirming the absence of carry-over. Blank plasma samples were collected in ethylenediaminetetraacetic acid (EDTA) from healthy donors recruited at the Blood Transfusion Center of the Children’s Hospital Bambino Gesù after obtaining informed consent. Drug-free plasma was recovered from whole blood samples by means of centrifugation at 3500 rcf for 5 min.

This method was validated according to the EMA guidelines for bioanalytical methods validation (European Medicines Agency. Guidelines on Bioanalytical Method Validation available at: https://www.ema.europa.eu/en/documents/scientific-guideline/guideline-bioanalytical-method-validation_en.pdf, accessed on 4 October 2022).

## 5. Conclusions

In this case report, we show the safe and effective use of cefiderocol in a pediatric patient with XDR-PA systemic infection and limited therapeutic options. To the best of our knowledge, this is the first case describing a pediatric patient treated with cefiderocol during ECMO and CRRT support. Despite the limitations of this case report, such as the lack of generalizability to broader populations and the absence of control groups for comparison, our findings suggest that extended infusion of the standard dose of cefiderocol in children subjected to ECMO and CRRT may be safely adequate for reaching the PK/PD target of efficacy. Although more studies are needed to assess the safety and PK profile of this drug in a larger pediatric population, cefiderocol can be considered as an off-label rescue therapy in a similar clinical scenario.

## Figures and Tables

**Table 1:** Vital signs and blood laboratory findings.

	Day 1	Day 7	Day 14	Day 28	Reference Values
Temperature (°C)	37.4	38	38.3	36.8	-
Hemoglobin (g/L)	11.3	11.7	8.3	9.5	10.50–15.50
Leukocytes ${(10}^{3}$/μL)	8.57	31.03	22.26	11.88	6.00–17.00
Neutrophils ${(10}^{3}$/μL)	5.99	27.15	16.10	3.130	2.00–9.00
Platelets ${(10}^{3}$/μL)	92	494	72	260	150–450
CRP (mg/dL)	1.45	7.84	13.07	0.15	<0.50
Procalcitonin (ng/mL)	0.39	3	8.13	0.1	<0.5
Creatinine (mg/dL)	0.2	0.2	0.16	0.2	0.16–0.39
BUN (mg/dL)	22	8	6	8	4–19
INR	1.28	1.87	1.51	1.15	0.86–1.22
D-dimers (µg/mL)	3.50	3.43	0.73	0.5	<0.50
Blood cultures	-	negative	PA-VIM	negative	negative
BAL cultures	-	PA-VIM	PA-VIM	negative	negative

CRP = C-reactive protein, BUN = blood urea nitrogen, INR = international normalized ratio, and PA-VIM = VIM-producing *P. aeruginosa*.

## Data Availability

Data are available from the corresponding author upon reasonable request.

## Supplementary Materials

The following supporting information can be downloaded at: https://www.mdpi.com/article/10.3390/antibiotics13010037/s1, Figure S1: Chromatograms obtained for Cefiderocol and IS.
