# Supplementary material for: The Use of Cefiderocol as Salvage Therapy in an Infant Receiving ECMO and Continuous Renal Replacement Therapy
Source: Antibiotics (Basel). 2023 Dec 30;13(1):37. doi: 10.3390/antibiotics13010037 (PMC10812431; doi:10.3390/antibiotics13010037)
Supplement: Supplementary file 1 [file antibiotics-13-00037-s001.zip › antibiotics-2757017-supplementary.pdf]

Case Report

The Use of Cefiderocol as Salvage Therapy in an Infant Receiving ECMO and Continuous Renal Replacement Therapy

Stefania Mercadante, Costanza Tripiciano, Lorenza Romani, Matteo Di Nardo, Gabriella Bottari, Bianca Maria Goffredo, Raffaele Simeoli, Isabella Guzzo, Laura Lancella, Charalampos Antachopoulos and Maia De Luca

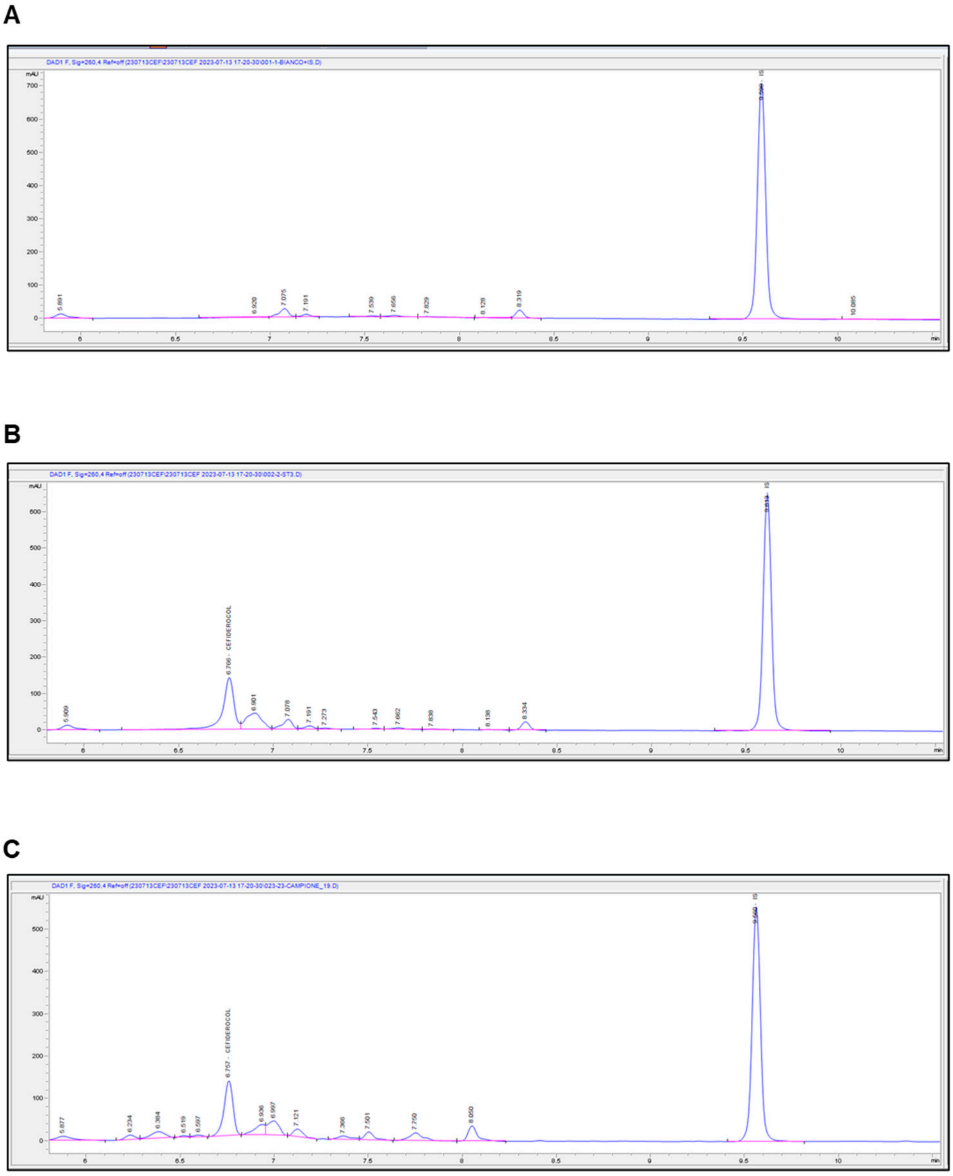

Figure S1. Chromatograms obtained for Cefiderocol and IS. (A) Fresh plasma blank sample spiked with IS; (B) Chromatogram for Cefiderocol calibrator 3 (50 µg/mL) spiked with IS; (C) Chromatogram for Patients' plasma sample. Cefiderocol concentration was measured at trough level (Ctrough). Relative response from the baseline (mAU) and the running time (min) are reported on the y- and x-axis, respectively. For each peak, compound name and retention time are displayed.
